# Assessing Lightning and Wildfire Hazard by Land Properties and Cloud to Ground Lightning Data with Association Rule Mining in Alberta, Canada

**DOI:** 10.3390/s17102413

**Published:** 2017-10-23

**Authors:** DongHwan Cha, Xin Wang, Jeong Woo Kim

**Affiliations:** Department of Geomatics Engineering, University of Calgary, Calgary, AB T2N1N4, Canada; cha8453@gmail.com or donghwan.cha@ucalgary.ca (D.C.); xcwang@ucalgary.ca (X.W.)

**Keywords:** association rule mining, cloud to ground (CG) lightning, hotspot analysis, wildfire hazard in Alberta

## Abstract

Hotspot analysis was implemented to find regions in the province of Alberta (Canada) with high frequency Cloud to Ground (CG) lightning strikes clustered together. Generally, hotspot regions are located in the central, central east, and south central regions of the study region. About 94% of annual lightning occurred during warm months (June to August) and the daily lightning frequency was influenced by the diurnal heating cycle. The association rule mining technique was used to investigate frequent CG lightning patterns, which were verified by similarity measurement to check the patterns’ consistency. The similarity coefficient values indicated that there were high correlations throughout the entire study period. Most wildfires (about 93%) in Alberta occurred in forests, wetland forests, and wetland shrub areas. It was also found that lightning and wildfires occur in two distinct areas: frequent wildfire regions with a high frequency of lightning, and frequent wild-fire regions with a low frequency of lightning. Further, the preference index (PI) revealed locations where the wildfires occurred more frequently than in other class regions. The wildfire hazard area was estimated with the CG lightning hazard map and specific land use types.

## 1. Introduction

Cloud to Ground (CG) lightning is a common meteorological hazard in Canada, and it is a leading cause of many types of fatalities, injuries, property damage, forest fires, and interruptions to business, as well as damage to almost every electrical or electronic system. About 9 to 10 lightning-related deaths and 19 to 164 injuries occur each year in Canada, costing between $3.6 and $79.2 million [1]. The lightning activity is a major natural ignition source for global wildfires and was parameterized as a driving factor to generate simulated wildfire model [2,3]. There is an average of 816 fires ignited by lightning each year. These fires cause an average of $16.4 million in property damage and between 3900 and 5300 insurance claims are estimated to be filed against lightning-related property damage (excluding fires) each year [4]. Around 75% of all forest fires are caused by lightning, with these fires accounting for about 85% of the total area burned in Canada [5]. CG lightning is the single largest cause of transients, faults and outages in electric power transmission and distribution systems in lightning-prone areas, and lightning is a major cause of electromagnetic interference that can affect all electronic systems [6].

Over the past few decades, a series of studies have focused on understanding lightning activity in many countries and various areas using different lightning location systems. The lightning location systems using natural lightning strikes to trees was presented by verification of lightning location accuracy in Finland [7]. The severity of lightning damages based on different sorts of trees was investigated with multiple parameters in Finland [8] and a hemlock-hardwood forest of the northern United States [9]. The relative proportions of intra-cloud to ground lightning ratio computed over the US in their combined analyses of NASA Optical Transient Detector (OTD) and National Lightning Detection Network (NLDN) Data Sets [10]. Spatial and temporal lightning flash density and occurrence are investigated in Canada [11,12,13,14,15], the United States [16], Europe [17], Estonia [18], the Mediterranean [19] and the Global scale [20,21]. The consistent results from all lightning pattern researches in different countries or areas are that the vast majority of lightning over land occurred during the warm months (May to October) with a strong peak in cloud to ground lightning between June and July and minimum seasonal lightning occurring in cold months (December to February). In addition, lightning strikes seem to be affected by the influence of the diurnal heating cycle. The distribution of lightning correlates well with the diurnal temperature cycle over land. The patterns can vary depending on topography, with the existence or nonexistence of water, weather conditions, and other specific properties as well. In fact, scientists are still unsure where and under what conditions lightning strikes occur. The research motive came from the question: If there are enough geo-coordinated lightning data, it may be possible to find a specific lightning pattern statistically.

There are not many published research outcomes for the relationship between CG lightning and land properties. These works examine possible impacts of land uses, soil types, elevation, vegetation cover, surface temperature, convective available potential energy (CAPE), etcetera, on lightning [22,23,24,25]. Usually they normalize the frequency of all the number of lightning strikes on a property and compare which classes within the property have more CG lightning frequency than other classes in each property. This study considers individual lightning record data with corresponding land properties (i.e., elevation, slope, land uses, soil types) to find a specific lightning pattern using association rule mining techniques. We can determine those association rules that highlight general trends in the database. Association rule mining is a very efficient way to find frequent patterns, but the efficacy of finding frequent CG lightning patterns using data mining techniques has not yet been researched to the best of our knowledge.

This paper has eight sections. In Section 2, we illustrate the general concept of association rule mining. We also summarize related association mining techniques and present their advantages and disadvantages for each algorithm. In Section 3, we explain the data we use for our research topic. In Section 4, we explain methodologies or theories relevant to our research, step by step. In Section 5, we verify the results of these methodologies. In Section 6, we verify the reliability of the outcomes. In Section 7, we suggest possible future applications for our data mining results and present one example of the potential application of our research, related to wildfires. In Section 8, we summarize the results of each section and draw conclusions from our research. In the discussion section, we present our research limitations and suggest a direction to improve this research in the future.

## 2. Background Knowledge of Association Rule Mining Techniques

### Concept of Association Rule Mining

Association rule mining is a data mining technology used to find frequent patterns that satisfy the user-specified factors in large databases. Agrwal and Srikant first came up with the idea of Association Rule Mining to analyze transactional databases and derive association rules [26,27]. The Apriori is a prototypical association rule mining algorithm [28] for mining frequent patterns for a large transactional dataset with a user specified single minimum item support count. The support count is an indication of how frequently the itemset appears in the dataset. However, the Apriori algorithm assumes that all items in the database are of the same nature or have similar frequencies. Therefore, if the minimum support is too high, those rules that include rare items are not found, and if the minimum support is too low for extracting both frequent and rare items, it causes a combinable explosion and high computation time. More recently, more advanced algorithms such as MSapriori [29], FP-Growth [30]. The CFP-Growth [31], and CFP-Growth++ [32] methods are proposed for making association rule mining more effective and efficient.

Among them, CFP-Growth++ introduces four pruning techniques to reduce search space so that we can save time. It uses a better principle to identify the item that never generates any frequent pattern through constructing a compressed MIS-Tree [31]. In addition, the CFP-Growth++ algorithm searches and finds suffix patterns that can generate frequent patterns at higher order only instead of searching all the possible patterns of suffix patterns in the MIS-Tree [32]. In our experimentation, we used the library called SPMF [33] for finding frequent patterns using CFP-Growth++.

## 3. Data Descriptions

### 3.1. Cloud-To-Ground (CG) Lightning Data

The Canadian Lightning Detection Network (CLDN) was established in 1998 and consists of over 80 lightning sensors distributed across Canada. The CLDN is part of the larger North American Lightning Detection Network (NALDN) that monitors lightning over most of North America. The NALDN is the largest lightning detection network in the world. The CLDN runs 24 h a day, 7 days a week, 365 days a year, and detects cloud-to-ground lightning strikes and a small percentage of cloud-to-cloud lightning. The CLDN is capable of detecting up to 45,000 lightning strikes an hour, although typically the maximum number of strikes per hour in Canada is less than 25,000. The CLDN’s lightning sensors determine the strength, polarity (positive or negative charge) and time of lightning strikes, all from the electromagnetic pulse the lightning produces [34]. The total number of CG lightning frequency and the size of data for Alberta from 2010 to 2016 are about 300,000 and 118 megabyte (MB) respectively.

### 3.2. Terrain Elevation and Terrain Slope Data

We obtained a terrain elevation data from Shuttle Radar Topography Mission (SRTM) [35] 1 Arc-Second Global data for our study area (the Province of AB, Canada). The absolute vertical accuracy of the elevation data will be 16 m (at 90% confidence). This radar system will gather data that will result in the most accurate and complete topographic map of the Earth’s surface that has ever been assembled. The unit of elevation is meters as referenced to the WGS84/EGM96 geoid. We then calculate terrain slope based on this digital elevation model using ArcGIS software (ESRI, Redlands, CA, USA) [36]. The terrain elevation and terrain slope data for Alberta, Canada were derived from this SRTM 1 Arc-second global data.

### 3.3. Land Uses Data

**Figure 1:**
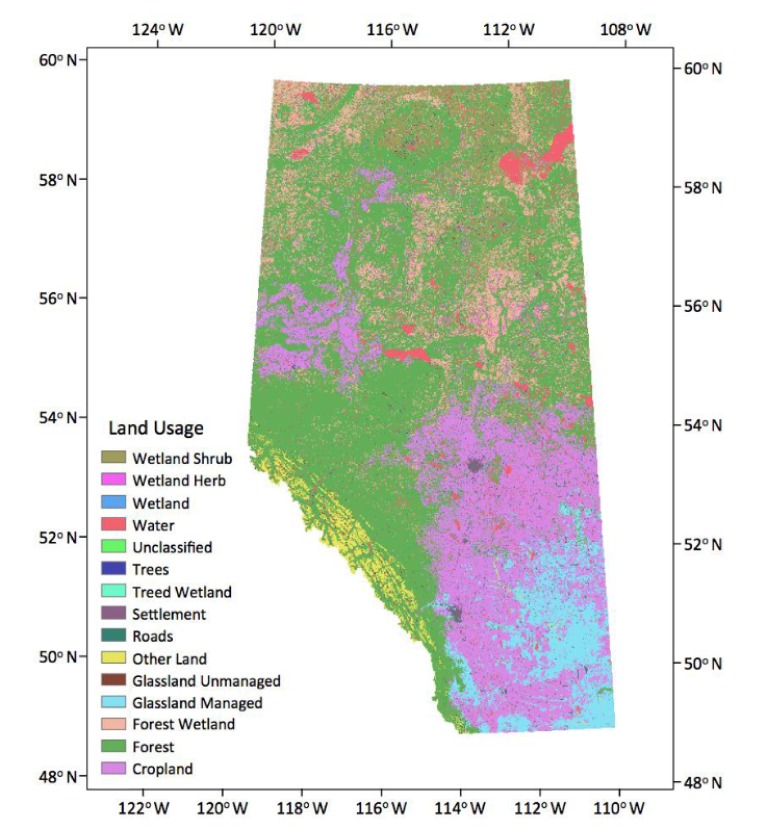
Land uses of Alberta (source: Natural Resources Canada, NRCan).

The Land Uses (LU) map (Figure 1) covers all areas of Alberta at a spatial resolution of 30 m. The LU classes follow the protocol of the Intergovernmental Panel on Climate Change (IPCC) and consist of: Forest, Water, Cropland, Grassland, Settlement and Other land (barren land, ice, rock and unclassified). The LU maps were prepared using existing source data, including a variety of land cover (LC) and crop maps and various topographic layers such as Buildings and Structures, Hydrography, Industrial and Commercial Areas, Transportation and Wetlands from the “Canada Vectors”, a digital cartographic product in vector format (CanVec) supplied by Natural Resources Canada (NRCan). Details about the data and their accuracy are given in Data Product specifications [37].

### 3.4. Soil Types Data

**Figure 2:**
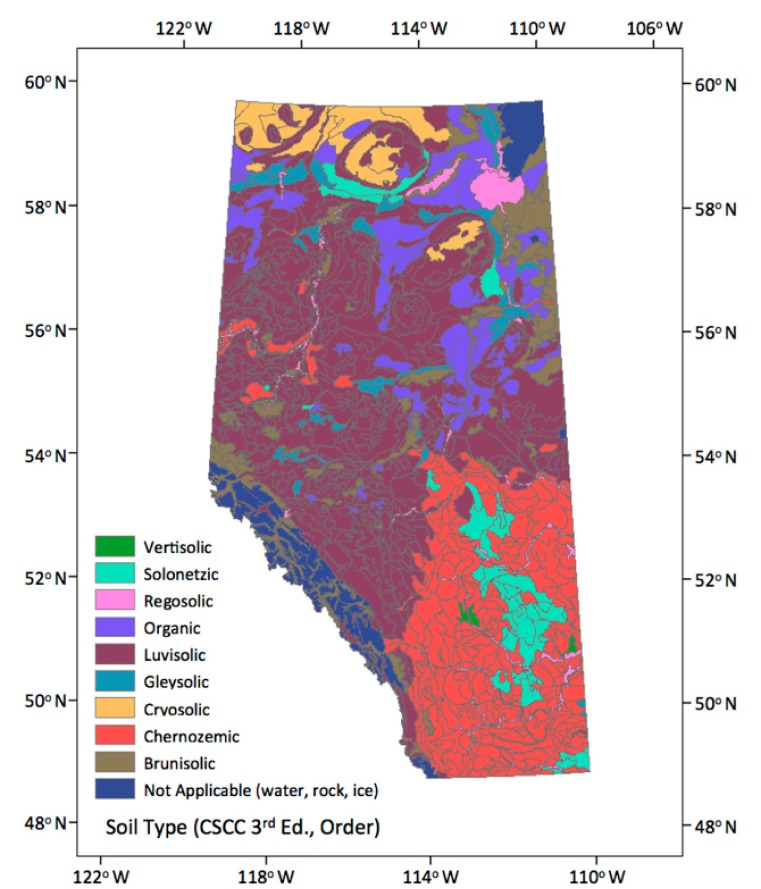
Soil Types (soil great group by the Canadian System of Soil Classification 3rd edition [38]).

Soil Landscapes of Canada data (SLC version 3.2, Soil Landscapes of Canada Working Group, Ottawa, ON, Canada Figure 2) is the latest revision of the Soil Landscapes of Canada, which was developed by Agriculture and Agri-Food Canada to provide information about the country’s agricultural soils at the provincial and national levels. SLCs provide GIS coverage that shows the major characteristics of soil and land for the whole country. The information is organized according to a uniform national set of soil and landscape criteria based on permanent natural attributes. They are based on existing soil survey maps that have been recompiled at 1:1,000,000 scale. SLC polygons may contain one or more distinct soil landscape components and may also contain small but highly contrasting inclusion components. We could get a distinct type of soil to identify the soil great group according to the Canadian System of Soil Classification, 3rd edition [38]. There are ten classes in the Canadian System of Soil Classification and the major levels used in the classification include: Order, Great Group and Subgroup. We used the first level (Order) within the System for distinct soil types [38,39].

### 3.5. Canadian National Fire Database (CNFDB)

The Canadian National Fire Database (CNFDB) is a collection of wildfire data from various sources; these data include fire locations (point data) and fire perimeters (polygon data) as provided by Canadian fire management agencies (provinces, territories, and Parks Canada) [40]. The properties of the wildfires are composed of agency information (province, territory, parks) illustrating which agency collected the fire data, wildfire occurrence dates, coordinates, fire size (hectares), cause of fire as reported by each agency, and fire types. These data will be used in the application section (Section 7) to demonstrate how the frequent pattern results from data mining can be utilized. We only use lightning-caused wildfire data, among all possible different causes of fire, in order to know the impact of the CG lightning strikes on wildfire outbreak.

## 4. Methodologies

### 4.1. Spatial Distribution of Lightning Strikes Activity (Hotspot Analysis)

**Figure 3:**
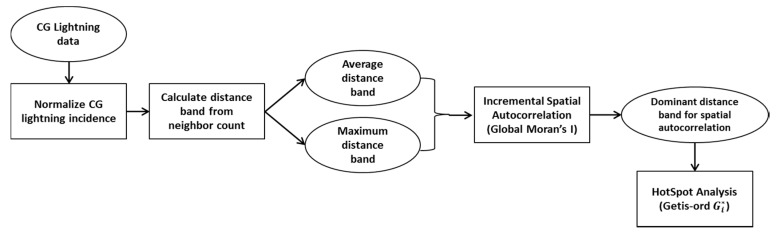
Flow chart of spatial lightning pattern analysis by statistical method.

The research purpose of this section is to investigate how CG lightning is distributed spatially over Alberta. We count CG lightning frequency in each municipal boundary to see which areas have more lightning. The study is based on the cloud to ground (CG) lightning data from 2010 to 2014. Hotspot analysis enables us to know not only where high-value regions cluster together but also where low-value regions cluster. Several processes precede actually carrying out hotspot analysis. The flow chart of the processes is below (Figure 3). We will summarize these processes first and then we will explain each step in greater detail. All processes are performed by ArcGIS 10.3 software (ESRI, Redlands, CA, USA) [36].

First, taking into account the cloud to ground (CG) lightning data for the study period from 2010 to 2014, we counted how many lightning strikes actually transpired in the predefined 84 Alberta municipalities. In order to overcome the size variations, we normalized the number of CG lightning flashes within a district by dividing it by the area of each district. The next step for carrying out hotspot analysis is specifying a threshold distance for neighboring features, which is called a neighborhood. The neighborhood consists of the features that are analyzed together in order to assess local clustering. The neighborhood is defined by a threshold distance band. To set the threshold distance band, the Incremental spatial autocorrelation tool [41] in ArcGIS 10.3 [36] is implemented. The Incremental spatial autocorrelation tool essentially finds appropriate distances where spatial clustering is most pronounced, by using incremental distances. However, we need to select both the starting distance and the distance increment values to implement the tool. The ‘Calculating distance band from neighbor count’ tool in ArcGIS 10.3 helps for this step. This tool tells us the minimum, average, and maximum distances when each feature has one neighbor. As we mentioned, we need to find a reasonable distance value that includes possible neighborhood features. Measuring spatial autocorrelation is one way to find the appropriate distance. Global Moran’s I [41] measures spatial autocorrelation based simultaneously on feature location and feature values. It can tell us whether a set of features is clustered, dispersed or random. To identify a spatial pattern of the normalized incidence of CG lightning data, we performed hotspot analysis (one of the spatial analysis tools in ArcGIS 10.3), which enabled us to identify the statistically significant hotspot where high incidence data values cluster together. The hotspot analysis calculates Getis-Ord $G_{i}^{*}$ statistic [42] as follows:

(1) $$G_{i}^{*}=\frac{\sum_{j=1}^{n}w_{i,j}x_{j}-\bar{X}\sum_{j=1}^{n}w_{i,j}}{S\sqrt{\frac{\left[n\sum_{j=1}^{n}w_{i,j}^{2}-{\left(\sum_{j=1}^{n}w_{i,j}\right)}^{2}\right]}{n-1}}}$$

where $x_{j}$ is the attribute value for feature *j*, $w_{i,j}$ is the spatial weight between feature *i* and *j*, and *n* is equal to the total number of features:

(2) $$\bar{X}=\frac{\sum_{j=1}^{n}x_{j}}{n}$$

(3) $$S=\sqrt{\frac{\sum_{j=1}^{n}x_{j}^{2}}{n}-{\left(\bar{X}\right)}^{2}}$$

$G_{i}^{*}$ Statistic is a Z-score so no further calculation is required. A Z-score is simply a measurement of standard deviation. This score serves as a basis for deciding whether we can reject the null hypothesis.

### 4.2. Temporal Distribution of Lightning Strikes Activity

There are slightly different maximum and minimum CG lightning occurrence densities in other studies but this temporal distribution is in agreement with several studies that have been conducted on global and local regional scales [11,14,16,17,18,20,21,23,25,43,44,45]. The results are similar to all lightning pattern research conducted in different countries or areas where the vast majority of lightning over land occurred during the warm months (May~October) with a strong peak in cloud to ground lightning (June~July) and minimum seasonal lightning occurrence in cold months (December~February). In addition, lightning strikes seem to be affected by the influence of the diurnal heating cycle. We assumed that the temporal distribution of CG lightning over Alberta was similar to that of other regions. To identify the temporal trend of the lightning over Alberta precisely, we divide all lightning occurrence counts by month and distribute each month’s count hourly. The resulting graphs, tables and interpretations are shown in the results section (Section 5.2).

### 4.3. Discovery of Frequent CG Lightning Patterns Using CFP-Grwoth++ Algorithm

We carried out experiments to identify the implicit lightning frequency patterns under given conditions. These given conditions are expressed in the four land physical properties (i.e., Elevation, Slope, Land Uses, and Soil Type). We subdivided each property into 10 classes of elevation data, 10 classes of slope data, 15 classes of land uses data, and 10 classes of soil type data. We used the Jenks natural breaks optimization method [46] for dividing elevation and slope data. The land uses classes follow the protocol of the Intergovernmental Panel on Climate Change (IPCC) and consist of: Forest, Water, Cropland, Grassland, Settlement and Other land (barren land, ice, rock and unclassified). There are distinct types of soil according to the Canadian System of Soil Classification [38]. Therefore, each lightning strikes record has those four different properties.

The main purpose of this experiment is to identify the relationships between those four properties and lightning strikes, or to see whether there are any particular frequent patterns and whether the frequent patterns are consistent throughout the study period. The main theory of CFP-Growth++ was implemented for this study but assigning minimum item support (MIS) values are modified in this study. Based on the original MIS functions, the user-specified MIS values are chosen by iterative calculation to generate the best result. However, the appropriate method to find the MIS value for each item differs slightly because we have an unequal range of coverage area for each item. Therefore, it is possible that a larger coverage area for one item has relatively more lightning strikes than a smaller coverage area for another, even though the larger area has a lower rate of lightning strikes per unit area. The modified MIS functions consider the characteristics of uneven coverage area size of each item and take the rare item problem into account as well. The modified MIS functions are as follows:

(4) $$\mathrm{MIS}\;(A)=\mathrm{Min}\;\{\mathrm{MIS}\;\left(a_{1}\right),\;\mathrm{MIS}\;\left(a_{2}\right)\ldots \;\mathrm{MIS}\;\left(a_{k}\right)\}$$

(5) $$\mathrm{MIS}(a_{i})=\{\begin{array}{cc} M\left(a_{i}\right) & \mathrm{if}\;M\left(a_{i}\right)>\mathrm{LS}\\ \mathrm{LS} & \mathrm{Otherwise} \end{array}$$

(6) $$M(a_{i})=\mathrm{Round}\;\mathrm{off}\;(\beta^{\prime }\;f\left(a_{i}\right))$$

(7) $$\beta^{\prime }=\frac{A_{a_{i}}}{A_{T}}$$

The value $f\left(a_{i}\right)$ is the actual frequency (or the support expressed in percentage of the data set size) of item $a_{i}$ in the data. The value LS is the user-specified lowest minimum item support allowed. β (0≤β≤1) is a parameter that controls how the MIS values for items should be related to their frequencies. The modified part is for β (0≤β≤1) value. Originally, the β value was a user-specified value and it is based on the iterative calculation to find the best MIS values. This is not only a non-standard way of choosing this MIS value, but it also fails to consider the different coverage area sizes. The value $A_{a_{i}}$ is the coverage area of item $a_{i}$, while $A_{T}$ is the total study area. By using this modified β value ($\beta^{\prime }$), we can fit for individual item characteristics. This modified MIS function can prevent possible problems, already mentioned above, when a larger coverage area for an item has relatively more lightning strikes than a different item’s smaller area, even though the larger area has fewer lightning strikes per unit area. The sequence result of non-modified MIS functions is in the larger coverage area of an item having fewer lightning strikes per unit area, which could cause it to be extracted as a frequent item. Therefore, we use the modified MIS function in this research.

## 5. Results

### 5.1. Hotspot Analysis (Spatial Distribution of CG Lightning)

**Figure 4:**
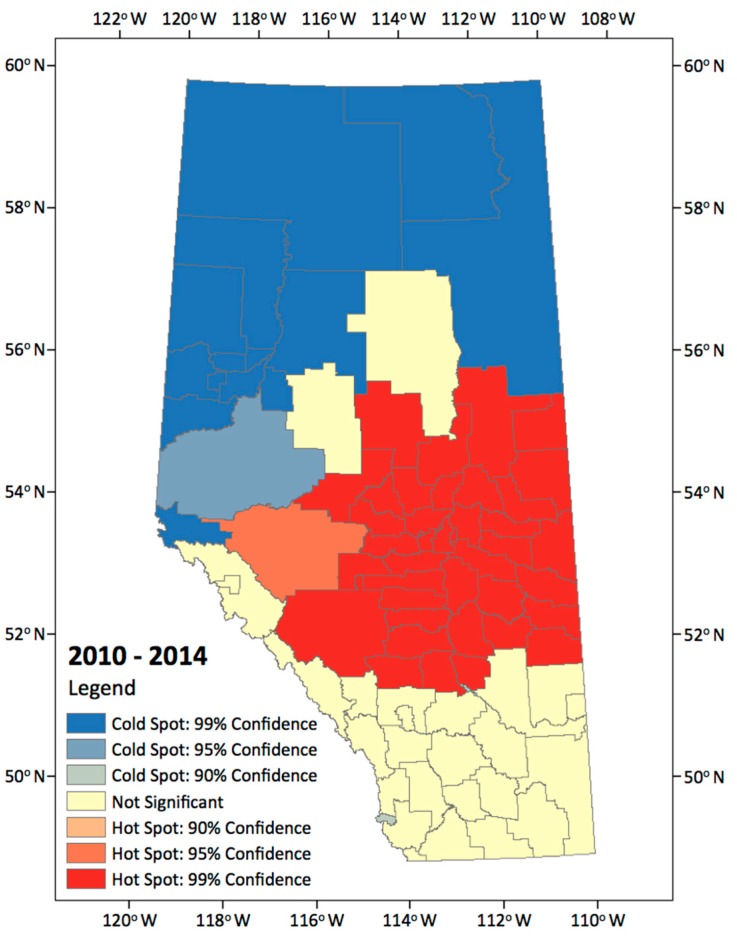
Hot and cold spot of annual lightning incidence rate for 2010–2014.

The hotspot analysis was conducted to identify statistically significant hotspot regions where high incidence CG lightning counts cluster together on predefined 84 Alberta municipalities. Using this method, we documented the spatial distribution of CG lightning, showing the hotspot analysis results for the entire combined study period (Figure 4).

This map shows clear spatial patterns indicating that most hotspot regions were located in central, central east, and south central regions of the study area, and coldspots are more typically found in northern areas. There are areas in which the lightning strikes occur frequently or rarely, and there are also areas in which locations containing statistically significant high or low number of lightning strikes values cluster together.

Since we used only the spatial location of lightning strikes to identify the presence or absence of spatial patterns, we cannot explain what types of topographical characteristics influence the spatial patterns. We are therefore limited in describing the precise relationship between lightning strikes and topographical characteristics (land properties). Therefore, we will use an Association Rule Mining technique to identify the kinds of factors, and their combinations, that are related to the locations where CG lightning flashed. We will do so using four different topographical characteristics, enabling us to find the frequent patterns that have the four different factors (land properties) in much of the lightning strikes data.

### 5.2. Temporal Distribution of CG Lightning Activitiy

**Figure 5:**
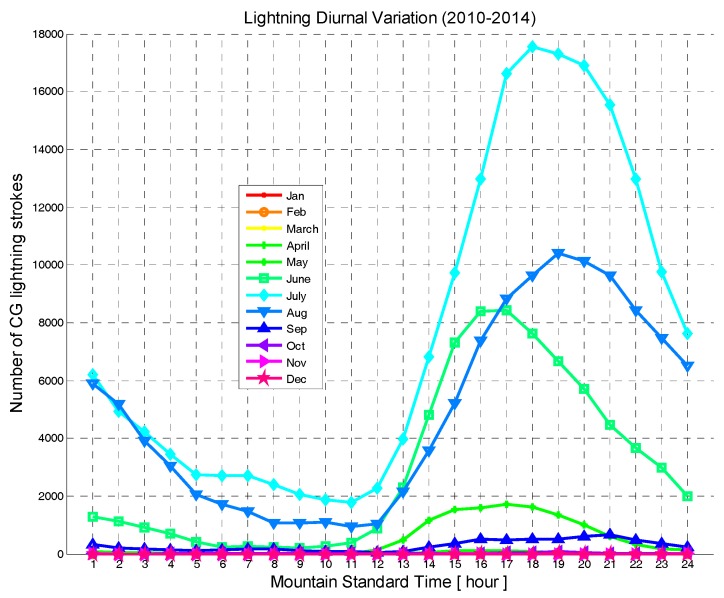
Average hourly lightning counts by month between 2010 and 2014.

Figure 5 shows the average hourly (Mountain Standard Time) lightning strikes counts by month in the Province of Alberta during the period of 2010 to 2014, and Table 1 shows their percentage of total lightning strikes counts. A total of 93.8% of annual lightning occurred in the warm months of June through August. Lightning activity was at its peak in July (45.7%). There are almost no lightning activities (0.0019%) in the cold months of December through February. Lightning strikes density steadily increases from about 10:00 local time (MST) to afternoon peaks occurring between 17:00 and 20:00 local time (MST). They then decline steadily to a morning minimum between 04:00 and 11:00 local time. Table 1 also shows that during the study period about 99.5% of all lightning strikes in the province of Alberta occurred between May and September.

### 5.3. Frequent CG Lightning Patterns Using CFP-5.3. Growth++ Algorithm

To find frequent patterns of CG lightning over Alberta, we used the CFP-Growth++ algorithm, but with the modified MIS function described in Section 4.3. We divided the frequent patterns of data mining results into five classes using the Jenks natural breaks optimization method. The classes are designated ‘High Risk’, ‘Risk’, ‘Moderate’, ‘Low Risk’ and ‘No Risk’. The average number of frequent patterns is about four hundred annually out of fifteen hundred given all possible combinations. We present ‘High risk’ and ‘Risk’ results for the entire research period (2010–2014) only here (Table 2).

We noticed the frequent patterns sorted in descending order of support counts are quite similar among all years of the study period, but we need to measure how similar patterns occurred statistically in order to prove that the CG lightning on our four different land properties is consistent or highly similar throughout all the study years. In addition, if we can find consistent CG lightning patterns on those land properties, we can use these frequent patterns for many applications in a variety of fields. In Section 6 (Verifications), we present a way of measuring similarity for our results sets and demonstrate the similarity value between two comparable results of all results sets. Each code number represents a category of land properties (i.e., Elevation, Slope, Land Uses, and Soil Types) and we explain all code numbers and their properties in Appendix A.

### 5.4. CG Lightning Hazard Maps

**Figure 6:**
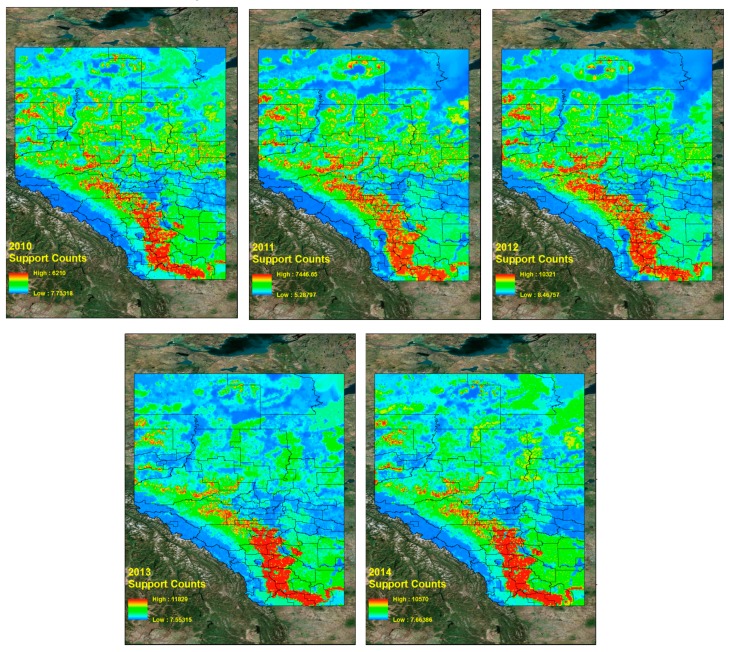
CG lightning hazard maps (2010–2014).

**Figure 7:**
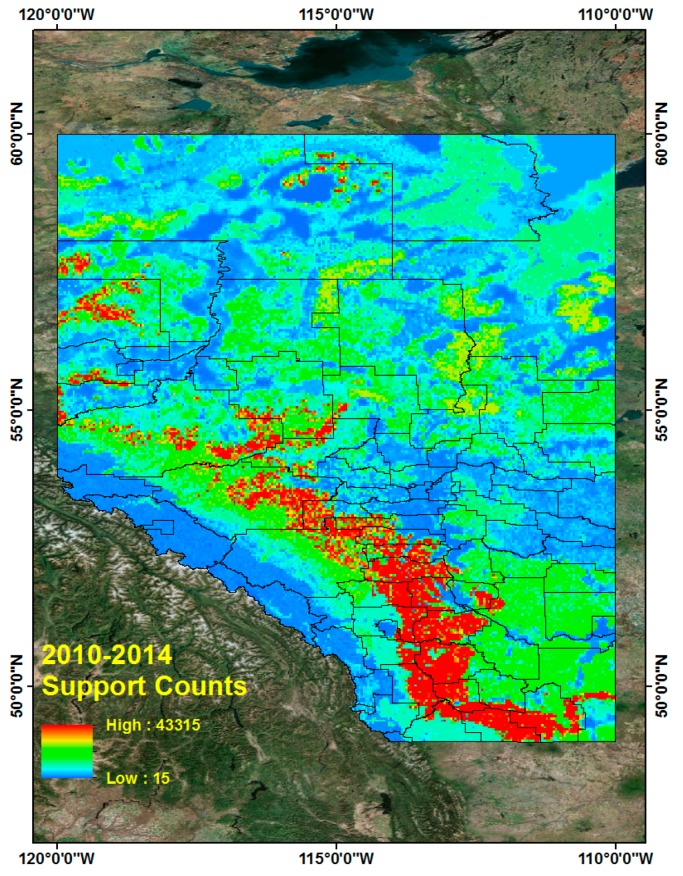
CG lightning hazard map for combined 2010–2014.

Based on these frequent tuples and support counts in Section 5.3, we generated CG lightning hazard maps and can illustrate which high CG lightning risk regions combine the four different land properties. The procedure for generating CG lightning hazard maps for the period from 2010 to 2014 is as follows. First, we collect all CG lightning data from 2010 to 2014. Each CG lightning data has patterns, which are sequences of four different land properties and their support counts. The CG lightning data is also geo-referenced data so that we can distribute the lightning points over Alberta. These distributed CG lightning points can show where CG lightning occurs more frequently and how particular combined land properties are more closely related with CG lightning strikes over Alberta. To make a continuous hazard map of CG lightning hazards, we use the Inverse Distance Weighted (IDW) interpolation tool [47,48] in ArcGIS 10.3 software [36]. Basically, the IDW interpolation determines cell values using a linearly weighted combination of a set of sample points. The weight is a function of inverse distance. Below are the resulting CG lightning hazard maps for each of 2010, 2011, 2012, 2013, 2014 (Figure 6) and the combined map for the period from 2010 to 2014 (Figure 7).

It is evident that high CG lightning risk areas are similarly positioned throughout the study years (2010–2014, Figure 6 and Figure 7).The high CG lightning risk areas are located in southern Alberta, along the east side of the Rocky Mountains. We can identify their corresponding land properties and their support counts (how much more frequent they are than other patterns) in frequent pattern results from the data mining results.

## 6. Verifications

### 6.1. Simiilarity Measure (Consistency Test) for Results

In this section, we measure the similarity between two frequent itemsets results from five years results. Similarity is a quantity that reflects the strength of relationship between two objects or two features. This quantity usually ranges between −1 and +1, or is normalized to between 0 and 1. Distance measures dissimilarity [49].

If the frequent patterns from the data mining algorithm have a consistency or high similarity throughout the years, we can conclude that the lightning data has specific patterns and these patterns have homogeneity. Our data mining result is multivariate categorical (nominal) data type. For continuous and bivariate data, the notion of similarity is relatively well established, but for categorical data and multivariate data, the similarity computation is not straightforward. For continuous data, the Minkowski Distance is a general method used to compute distance between two continuous multivariate points [50]. In contrast, we are not measuring a similarity between two individual results for a year; rather, we are measuring the similarity between two different sets. In other words, we need to measure the similarity between two clusters (sets) composed of a discrete (nominal) multivariate data type. There are many criteria in the literature (see [51,52,53,54]) for comparing two sets or groups with a view to measuring their similarity. However, there are some limitations in that literature. For instance, the literature [52] may not permit overlapping or joint clusters (sets), or it [53,54] may consider measuring similarity for overlapping clusters, but assume that a cluster does not contain duplicates. Given these limitations, the conventional methods in the literature are not applicable to our frequent itemset results. We explain our data type and structure first (Table 3) and propose an improved method of measuring similarity to overcome these limitations. The variables (Var1~Var4) are independent of each other and describe each land property. As we can see here, there are many joint (overlapping) frequent tuples within each result. For example, in the 2010 results (Table 3, left), the Elevation property 5 in the first pattern is duplicated in the second. Furthermore, the support count of each frequent pattern implies that there are duplicates. There are more details about the limitations in measuring similarity between two groups in the literature [52,53,54].

To take care of those limitations, we use a Best Match algorithm [53], which takes as its input the two cluster sets, $C_{1}$ ($S_{i=1,\ldots ,n}$) and $C_{2}$($S_{j=1,\ldots ,m}$), and a set difference measure. We modify this algorithm by converting support counts into a ranking score to assign a weight value for each frequent pattern. The Best Match algorithm determines how well the frequent patterns reflect each other. Specifically, for each frequent pattern ($S_{i}$) ∈ $C_{1}$, we can compute its best representative in $C_{2}$ by:

(8) $$S\left(S_{i},\;S^{\prime }\right)=\underset{j=1,\ldots ,m}{\max}S\left(S_{i},S_{j}^{\prime }\right)$$

This formula is slightly different from the original Best Match algorithm for finding the minimum of dissimilarity (distances) among all comparable patterns, but this formula (Equation (12)) finds maximum similarity among all comparable patterns. To measure the similarity between two nominal frequent patterns, we used Jaccard’s coefficient, which measures asymmetric information on variables. Since our variables are in the form of categorical (nominal) data, we cannot measure the variable in a quantitative way. We assigned a range of numeric indices to represent each item of variables. This is called consistent labeling. To calculate similarities or distances between two data sets represented by nominal variables, we need to convert these nominal variables into binary dummy variables that have binary values. A binary dummy variable is one that takes the value 0 or 1 to indicate the absence or presence of some categorical effect.

We can calculate the binary distance between the two binary dummy variables using Jaccard’s coefficient (Similarity) formula given by:

(9) $$Sim_{i,j}=\frac{p}{p+q+r}$$

where *p* is the number of variables that are positive (1) for both objects; *q* is the number of variables that are positive (1) for the ith object and negative (0) for the jth object; and *r* is the number of variables that are negative (0) for the ith object and positive (1) for the jth object.

We compute how well $C_{2}$ can be represented by $C_{1}$ by summing similarity coefficients from each member of $C_{1}$ to its respective best representative in $C_{2}$. We can make this similarity coefficient symmetric by also summing the similarity coefficients from every member of $C_{2}$ to its corresponding best representative in $C_{1}$ [53].This gives the final symmetric measure:

(10) $$\mathrm{Sim}\left(C_{1},C_{2}\right)=\overset{n}{\underset{i=1}{\sum }}\underset{j=1,\ldots ,m}{\max}\mathrm{Sim}\left(S_{i},S_{j}^{\prime }\right)+\overset{m}{\underset{j=1}{\sum }}\underset{i=1,\ldots ,n}{\max}\mathrm{Sim}\left(S_{j}^{\prime },S_{i}\right)$$

**Figure 8:**
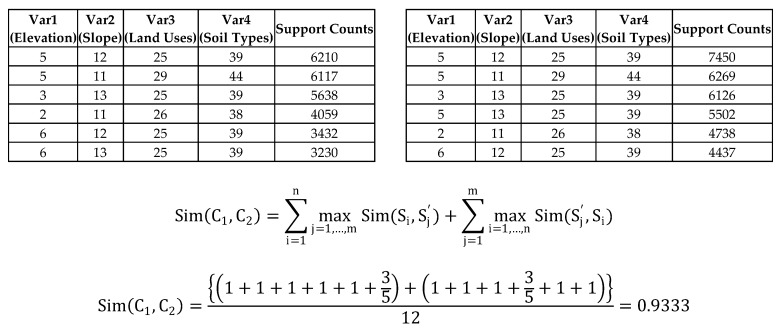
Example of Similarity Measure without Weight factors (Support Counts).

We can normalize this similarity using the number of clusters $\left|C_{1}\right|+\left|C_{2}\right|$ to make the final similarity score range from 0 to 1. There is one more thing that we need to consider about this similarity measure on our data sets. As we mentioned in the discussion of our data type, we have support counts for each frequent itemset. Therefore, we need to consider these support counts when we measure the similarity; we can do so by assigning weight values according to the support counts. To clarify, here is one example (Figure 8).

Table 3 is frequent itemset results for each year and they are sorted in descending order based on support counts. If we give rankings by its support count, the data look like below (Table 4).

The similarity coefficient in Figure 8 is about 0.9333 but this method ignores the very important information added by the support count for each frequent pattern. The support count of each frequent pattern illustrates how a frequent pattern is more important (frequent) than another within the result itself. The sheer volume of its support count can vary depending on the absolute size of input data. Therefore, when we measure similarity between two clusters, we need to focus on the relative order of priority for each cluster and consider rank differences between the two patterns as a weighting factor. To illustrate a potential influence from these rankings, we will give you an example using Table 4. When we measure maximum similarity based on $S_{4}$ ∈ $C_{1}$ against $S_{j=1,\ldots ,6}^{\prime }$ ∈ $C_{2}$, the maximum similarity can be found in $S_{5}^{\prime }$; Their Jaccard’s coefficient value is 1 even if there is a difference of one rank. When there is a rank difference between two comparable frequent patterns, we need to consider how their ranks differ and deduct their similarity coefficient value, which depends on the differences, by assigning a weight factor ($W_{i,j}\in \left[0,\;1\right]$). In this way, the similarity coefficient value of $S_{4}\;\left(\mathrm{rank}\;4\right)$ against $S_{5}^{\prime }\;\left(\mathrm{rank}\;5\right)$ must be less than 1 because there is a difference of rank. In other words, we would only set the similarity number at 1 (maximum similarity value), when each frequent pattern from each cluster is the same and their rank order is the same. Therefore, we contemplate the conformity of each pattern and set a corresponding ranking order. The following formula is the expanded scope of the Best Match algorithm [53]:

(11) $$\mathrm{Sim}\left(C_{1},C_{2}\right)=\overset{n}{\underset{i=1}{\sum }}\underset{j=1,\ldots ,m}{\max}W_{i,j}\mathrm{Sim}\left(S_{i},S_{j}^{\prime }\right)+\overset{m}{\underset{j=1}{\sum }}\underset{i=1,\ldots ,n}{\max}W_{i,j}\mathrm{Sim}\left(S_{j}^{\prime },S_{i}\right)$$

where the weight factor is given by:

$$W_{i,j}=\{\begin{array}{c} {\left(\frac{\left|C_{2}\right|-\left|r_{j}-r_{i}\right|}{\left|C_{2}\right|}\right)}^{p}\;\mathrm{if}\;\left|C_{2}\left|\geq \right|C_{1}\right|\\ {\left(\frac{\left|C_{1}\right|-\left|r_{j}-r_{i}\right|}{\left|C_{1}\right|}\right)}^{p}\;\mathrm{otherwise} \end{array}$$

$r_{i},r_{j}$ : Rank order of a frequent pattern respectively. p∈[0,1].

**Figure 9:**
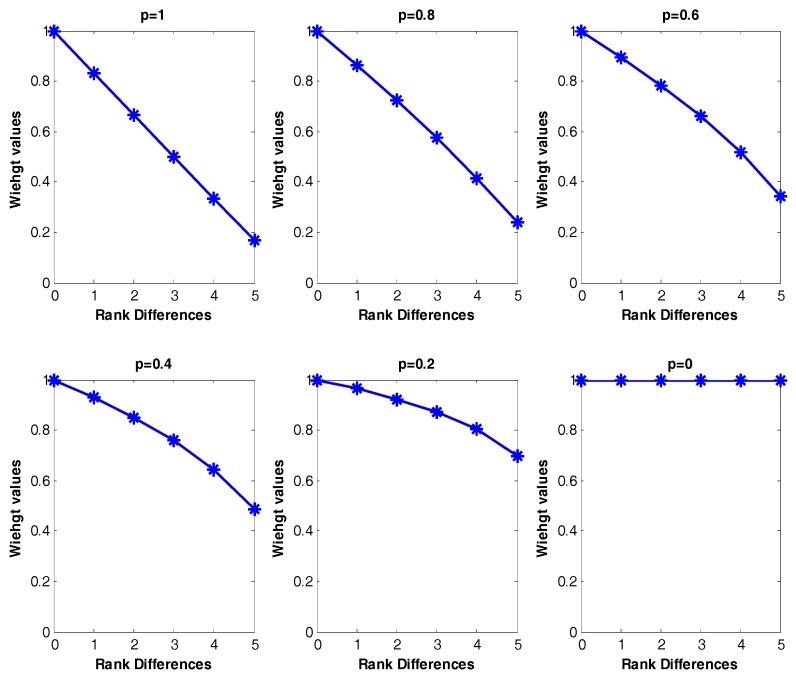
Weight values differences (from 0 to 1) by different *p*-values.

The weight value is from 0 to 1 and the *p* value in the weight factor may be estimated by a decision maker using the weight of the most important criterion. *p* = 1 indicates decreasing weight values with same interval and *p* = 0 indicates equal weight values. Basically, *p* values (0 < *p* < 1) make the weight values proportionally decrease as the rank differences increase. For example, when we measure the weight values for two frequent patterns results of Table 4, the possible rank differences are from 0 to 5 and the number of clusters are six for both $\left|C_{1}\right|$ and $\left|C_{2}\right|$. The larger the rank differences between two frequent patterns from each cluster, the smaller the weight values generated; the degree of decrease for the weight values is based on the user-specified *p*-value. As *p* decreases from 1 to close to 0 (non-zero) in Figure 9, the weight value slowly decreases as the rank differences increase. If the *p* value is 0, this is equivalent with the original Best Match algorithm [53], ignoring rank differences of the two frequent patterns. Figure 9 shows the possible weight distribution by different *p*-values for Table 4.

**Figure 10:**
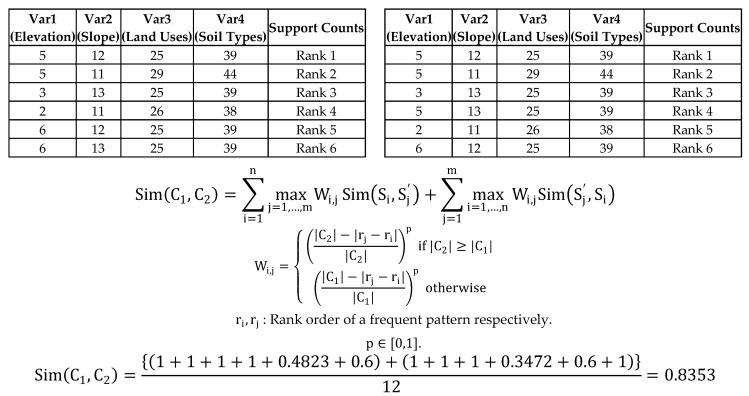
Example of Similarity Measure with Weight factors (Support Counts), *p*-value = 1.

If we calculate the similarity by considering weight values for rank differences based on the Best Match algorithm with *p* = 1 for a given example (Table 4), the similarity measurement result can be calculated as below (Figure 10).

The Best Match algorithm is modified by assigning weight factors based on rank similarity results in the 0.8694 similarity coefficient value (Figure 10), which is lower than 0.9333 (Conventional Best Match algorithm, Figure 8). This is because there are rank dissimilarities between two possible comparison frequent tuples from two clusters respectively. We compute all the similarity coefficient values between two frequent pattern data sets (clusters) throughout five results, from 2010 to 2014, with different *p* values (0<p≤1) that can be chosen by the decision maker. Listed below are the final similarity coefficient results (Table 5) using the modified Best Match algorithm by assigning weight factors based on rank similarity.

The minimum similarity coefficient value is about 0.85 (85%) and the maximum similarity coefficient value is about 0.95 (95%), with different *p* values for all possible combinations of two frequent tuple sets of two years. These results that there are high correlations between the two frequent sets results of all possible two year’s results; this means that there are quite consistent similarities or consistent patterns for all years. In other words, there are quite consistent frequent lighting patterns for the four different land properties (i.e., Elevation, Slope, Land Uses, and Soil Types).

### 6.2. Additional Verification Process by Comparison between 2010–2014 and 2015–2016 Data Mining Results

In the early stages of our research, we had only 2010–2014 CG lightning data and carried out all the related experiments with this data. It is highly desirable that we use the latest dataset (2015–2016) to see whether our experimental results (2010–2014) remain reliable when we compare them to results for the latest dataset. We conducted Association Rule Mining with the more recent dataset to test how consistent these new results are with the old results. We did so by measuring similarity values and comparing the two CG lightning hazard maps from the two different periods (2010–2014 and 2015–2016). Here are the two frequent pattern results, 2010–2014 and 2015–2016 (Table 6), using Association Rule Mining (CFP-Growth++ algorithm). We also divided the frequent patterns of data mining results into five classes (‘High Risk’, ‘Risk’, ‘Moderate’, ‘Low Risk’ and ‘No Risk’) using the Jenks natural breaks optimization method. We present ‘High risk’ and ‘Risk’ results only.

We recognize that the frequent patterns for 2010–2014 and for 2015–2016 are quite similar. We also measure the similarity coefficient value between these two results to demonstrate that they have a similar quantitative numerical value. The methodology of measuring similarity among Association Rule Mining results is described in Section 6.1. We measure the similarity by changing parameter *p*-values (*p* = 1, 0.8, 0.6, 0.4 and 0.2). The similarity results (Table 7) are described below:

The above results show that there is a very high correlation between the two frequent sets results for 2010–2014 CG lightning and 2015–2016 CG lightning. In other words, there are quite consistent similarities or patterns for all years. 

**Figure 11:**
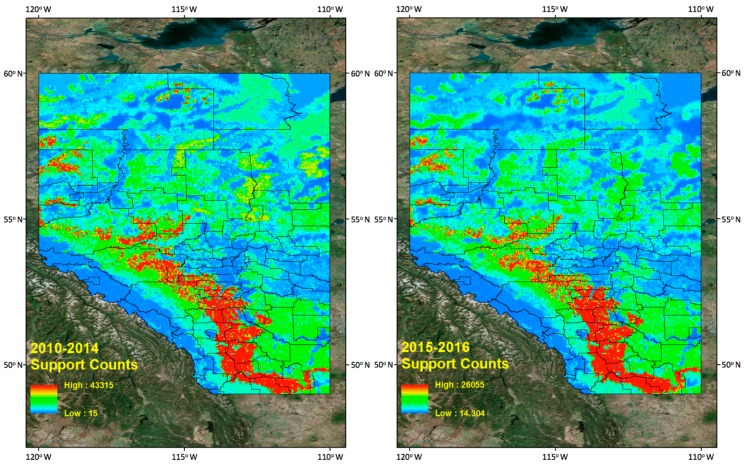
CG lightning hazard maps. (**Left**) 2010–2014, (**Right**) 2015–2016.

In Section 6.1, we only measure a similarity value for two individual years, but the combined frequent patterns from 2010 to 2014 and 2015 to 2016 and their two similarity values show much greater similarity than do the individual year’s results. The similarity measurement results between 2010–2014 and 2015–2016 tells us there are quite consistent frequent lighting patterns for the four different land properties (i.e., Elevation, Slope, Land Uses, and Soil Types). In addition, we generated 2010–2014 and 2015–2016 CG lightning hazard maps (Figure 11) to see if the new 2015–2016 CG lightning hazard areas have spatial distribution of the lightning on four land properties consistent with that of 2010–2014 CG lightning hazard areas. The methodology for generating hazard maps is described in Section 5.4.

As mentioned before in Section 5.4, most recognizable high CG lightning risk areas are located in southern Alberta along the east side of the Rocky Mountains. We can find their corresponding land properties and their support counts in frequent pattern results from the data mining results. We find a very similar spatial distribution of the CG lightning hazardous area for the new dataset (2015–2016) when we compare it with the 2010–2014 dataset.

### 6.3. Comparsion of CG Lightning Hazard Map (2010–2014) with Actual Raw CG Lightning Data (2015–2016)

The CG lightning hazard map for 2010–2014 based on data mining results is divided into 20 classes that occupy the same size of area. The risk values of the map are sorted in ascending order for these 20 classes, where each class has about 5% of the total study area (Table 8). The hazard map based on the data mining results and the unprocessed raw CG lightning data are independent of each other. What we want to know is whether the higher CG lightning risk classes have more actual CG lightning from 2015 to 2016. In answering this question, we can verify whether the result patterns from data mining techniques are reliable.

Based on our assumptions, the higher the risk level of the class, the greater the count of 2015–2016 lightning data that must appear. The actual CG lightning strikes in each class quantify how many lightning strikes flash within that class. Table 9 records all relevant data.

**Figure 12:**
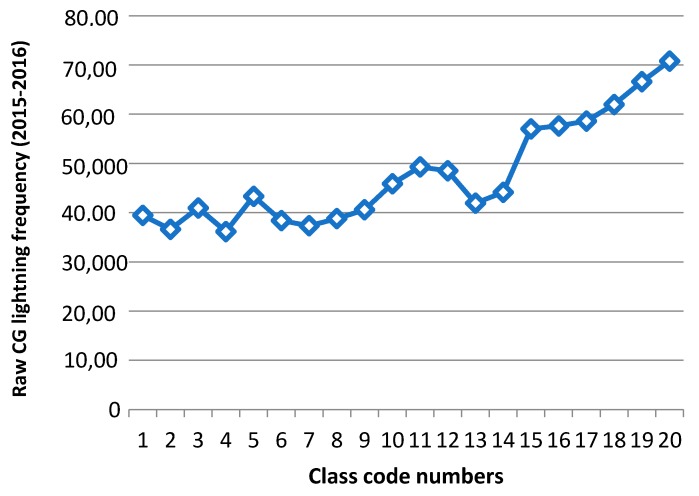
CG lightning frequency (2015–2016) into classes of hazard map classes (2010–2014).

Table 9 shows the actual CG lightning distribution (2015–2016) for classes (by class code number) of CG hazard maps (2010–2014) derived from data mining results. If we look at the CG lightning count values in Table 9, the general trend shows that raw CG lightning counts increased when the hazard map ranges (code numbers) increased. We can check the lightning frequency graph (Figure 12) of raw CG lightning sorted into classes based on the hazard map results (2010–2014).

We calculated a hazard map based on the data mining results. It consisted of two parts: four different land properties and their supports. We made a continuous CG lightning hazard map over Alberta, Canada based on those mining results. The unprocessed raw CG lightning data’s general frequency trends from 2015 to 2016, classed into the hazard map from 2010 to 2014, increases as the risk classes (class code numbers) increase. This trend is notably more dominant in classes ranging from code number 13 to 20. On the other hand, there are still quite a significant number of CG lightning strikes in lower risk areas. This may suggest some research limitations.

**Figure 13:**
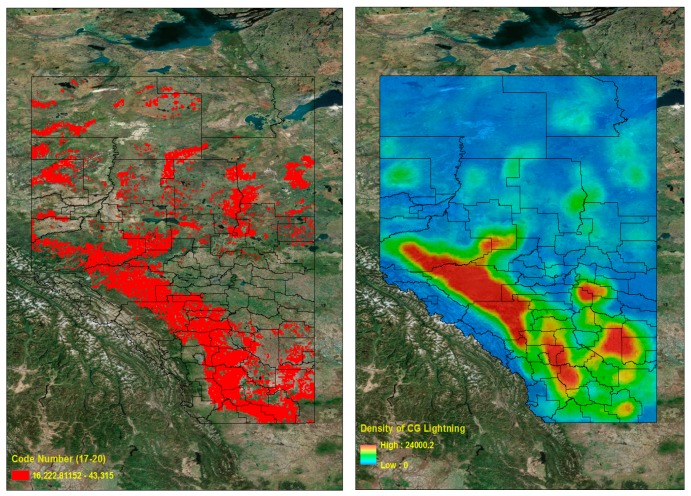
High risk CG lightning area (Code number 17–20) in the hazard map (2010–2014) (**Left**) and CG lightning density map (2015–2016) (**Right**).

We limited our analysis to the four land properties (i.e., Elevation, Slope, Land Uses, and Soil Types), but there could be many other triggers for lightning flashes on the surface. Despite the limited assumptions we set, the general trends of CG lightning for 2015–2016, classed on the hazard map for 2010–2014, show that higher risk areas on the hazard map have more actual CG lightning. We can also check these patterns by generating a density map of CG lightning. The lightning density map is based on the calculation of a magnitude-per-unit area from the lightning point features that fall within a neighborhood. We used the Point Density tool [55] in ArcGIS software [36] to make this density map (Figure 13 (Right)).

**Figure 14:**
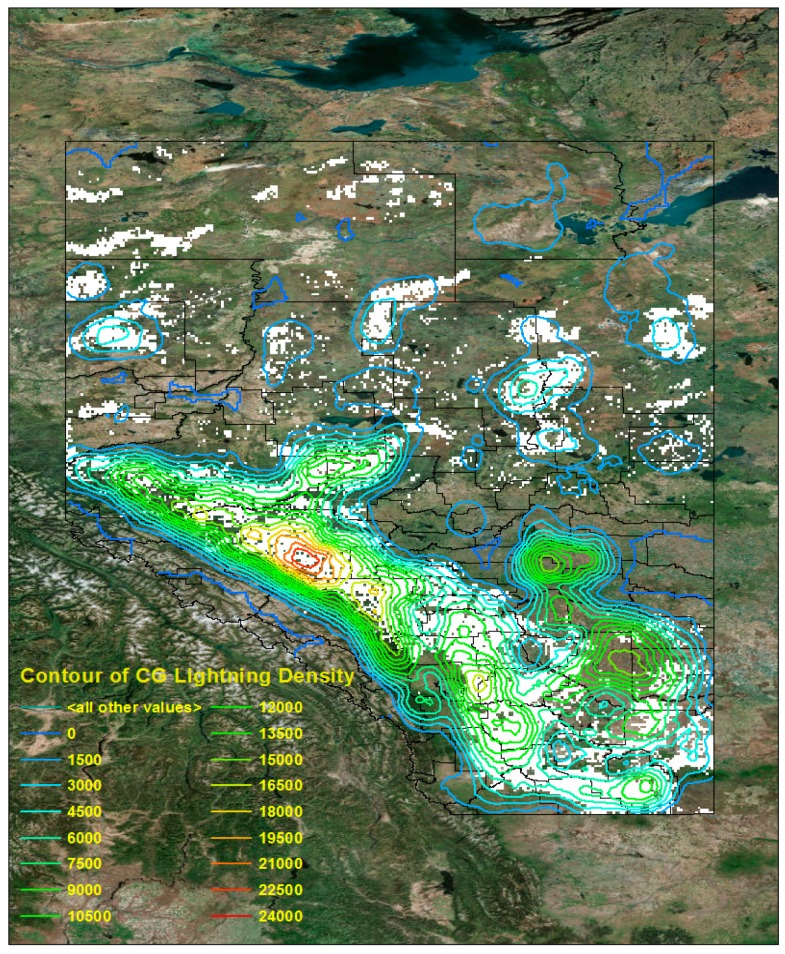
Overlaying image of CG lightning density (2015–2016) and high risk CG lightning area in the hazard map (2010–2014).

We divided the Province of Alberta into two clusters to look at the regions of high CG lightning density. The first cluster includes higher lightning risk areas (code numbers 17–20), which cover 20% of the total study area (Figure 13 (Left)), and the second cluster includes lower lightning risk areas (code numbers 1–16), which cover 80% of the total study area. In addition, we can check Table 9 for the corresponding percentage of the CG lightning counts from 2015 to 2016, which for these two clusters (groups of code numbers) are 26% and 74%, respectively. In Figure 14, the area marked in white is the same as the high risk (first cluster) regions in Figure 13 (Left). The contour lines describe a density map of raw CG lightning for 2015–2016. We can see that most high CG lightning density areas are located in the higher risk regions (code numbers 17–20, Figure 13 (Left)). In conclusion, we can verify by quantitative measurement from Table 9 and visual interpretation from Figure 14, that the higher risk areas on the hazard map have the more numerous and more densely occurring CG lightning flashes.

## 7. Applications (Analysis of Wildfire Hazardous Regions Based on the CG Lightning Hazard Map)

This section seeks to find the relationship between wild fires and CG lightning strike patterns derived from data mining processes (and CG lightning strike data). We extracted the wild fire data described in Section 3.5 to isolate only the fires in Alberta, and to count how many wild fires occurred in conjunction with particular land uses (Table 10). 

In Alberta, from 2010 to 2014, approximately 93% of total wild fires were caused by lightning on Forest, Forest Wetland, and Wetland Shrub land types (shaded boxes in Table 10). To better see how the wild fire points are geographically distributed, we scattered the wild fire data points on the hazard map.

**Figure 15:**
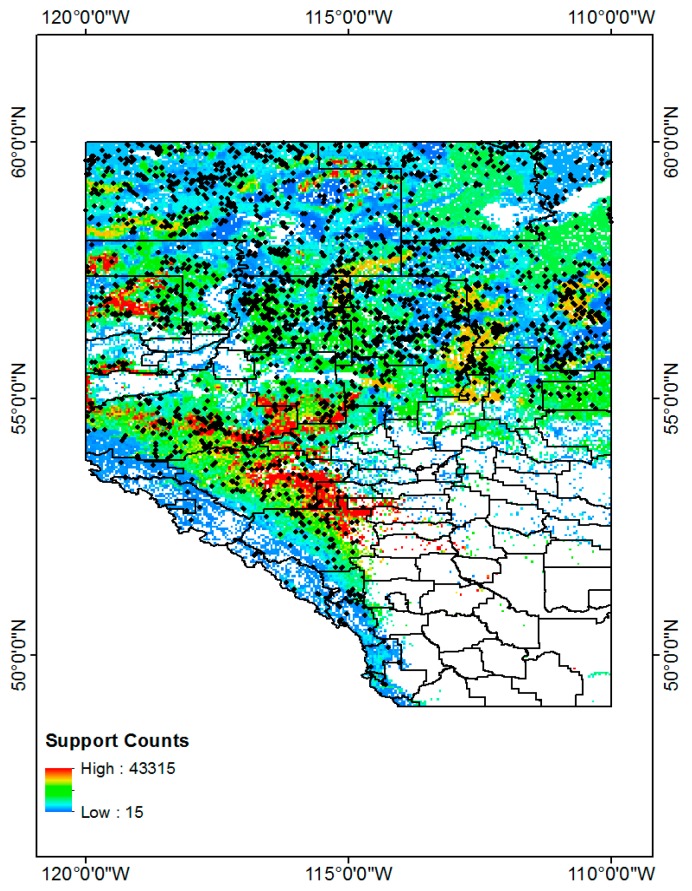
Wildfire locations on the CG hazard map only with Codes 25, 26 and 33 (Forest, Forest Wetland and Wetland Shrub) regions.

A higher risk of CG lightning does not always imply a higher risk of wildfires caused by CG lighting. There may be areas with more frequent CG lightning but lower frequent wildfires because of some other conditions, including weather and the strength of CG lightning strikes. Therefore, we limited the CG lightning hazard map to three dominant Land Uses classes, to reflect the finding that 93% of total wild fires caused by lightning occurred on Forest, Forest Wetland, and Wetland Shrub (Table 10). Figure 15 contains the hazard map overlaid with wildfire data.

**Figure 16:**
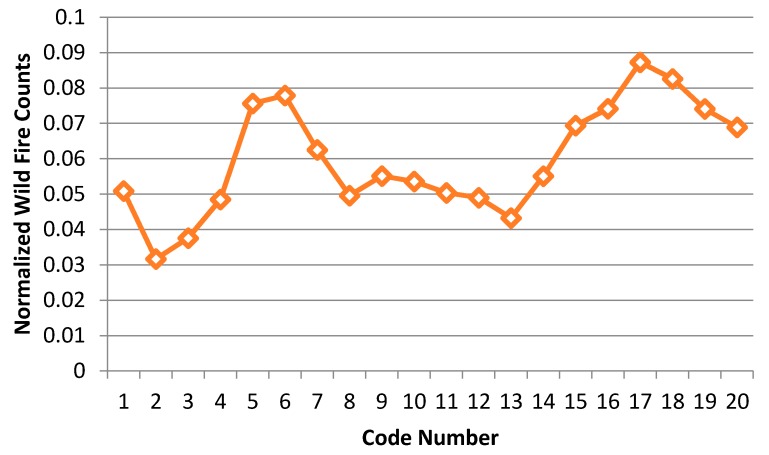
Normalized wildfire frequency (2010–2014) for each code number.

We divided the CG lightning hazard map (Figure 15) into 20 classes, based on the same criteria used in Table 9, then counted the wildfire frequency for each class. The code numbers in Table 11 are labels that represent each class, where higher code numbers correspond to higher CG lightning risk areas. We also divided the wildfire counts according to the size of the code number so that we could normalize the counts. This normalized wildfire count is designed to compensate for the problem of different code number sizes. Figure 16 is a normalized wildfire frequency graph that shows the trends visually.

The trend of the normalized wildfire counts in Table 11 and Figure 16 contains two peaks as the code numbers (risk labels of the hazard map) change. We divided the graph into two groups. First, the counts are increasing from code number 2. Then there is a peak at code number 6 and the counts start decreasing from this peak to code number 8. Second, the counts are increasing again from code number 13. Then there is a peak at code number 17 and they start decreasing again from the peak to code number 20. Given these ups and downs, it is hard to say that the risk of CG lightning must increase with the occurrence of wildfires. We can identify four possible cases to describe and account for this shifting relationship between CG lightning and wildfire:

1) The regions have a relatively high incidence of CG lightning strikes, but wildfires caused by lightning rarely occurred.
2) The regions have a relatively high incidence of lightning strikes and wildfires, and fires caused by lightning occurred frequently.
3) The regions have a relatively low frequency of CG lightning, but wildfires caused by lightning nevertheless occurred frequently.
4) The regions have a relatively low frequency of CG lightning, and wildfires caused by lightning rarely occurred.

The data (and two of the cases bolded above) suggest that there might be other triggers for wild fire by lightning, such as weather conditions and the particular characteristics of the lightning even when there are few lightning strikes. If the objective of this study is to research wildfire hazard, then it is the second and third cases that attract our interest. Therefore, we need to distinguish between those two areas having a relatively large incidence of wildfires and other study regions. To do so, let us start by demonstrating the notion of relative to know which areas have relatively more incidences of wildfires than others. The notion of relative in here can be calculated by Preference Index (PI) as follows:

(12) PI=WFnAnWFtAt

where ${\mathrm{WF}}_{n}$ is wildfire counts for a code number “n”, ${\mathrm{WF}}_{t}$ is the value of total number of wildfire counts for study area, $A_{n}$ is the area size for a code number “n”, and $A_{t}$ is the total size of whole study area. WF and A are the number of wildfires and the area size, respectively; the subscript n indicates the given category (code number) in the CG lightning hazard map; and the subscript t represents total area. The PI~1 would mean that the percentage of wildfires over each category of the CG lightning hazard map is equal to the percentage of wildfires over the entire study area. Therefore, if the PI is higher than 1, we can consider the wildfires relatively frequent compared to other categories in the CG lightning hazard map. In Table 12, we record PI for each code number from 1 to 20.

**Figure 17:**
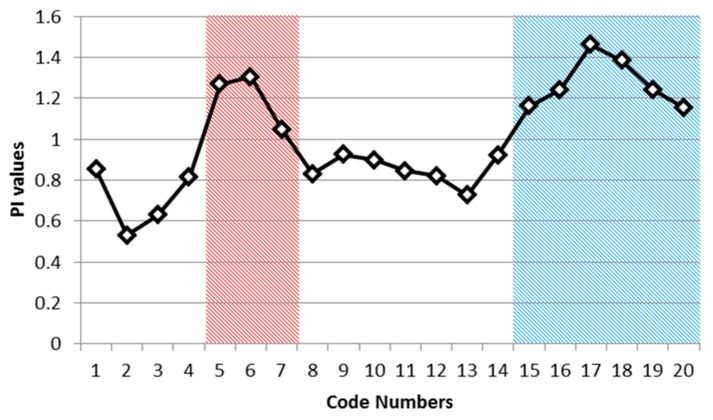
High risk wild fire area based on PI.

**Figure 18:**
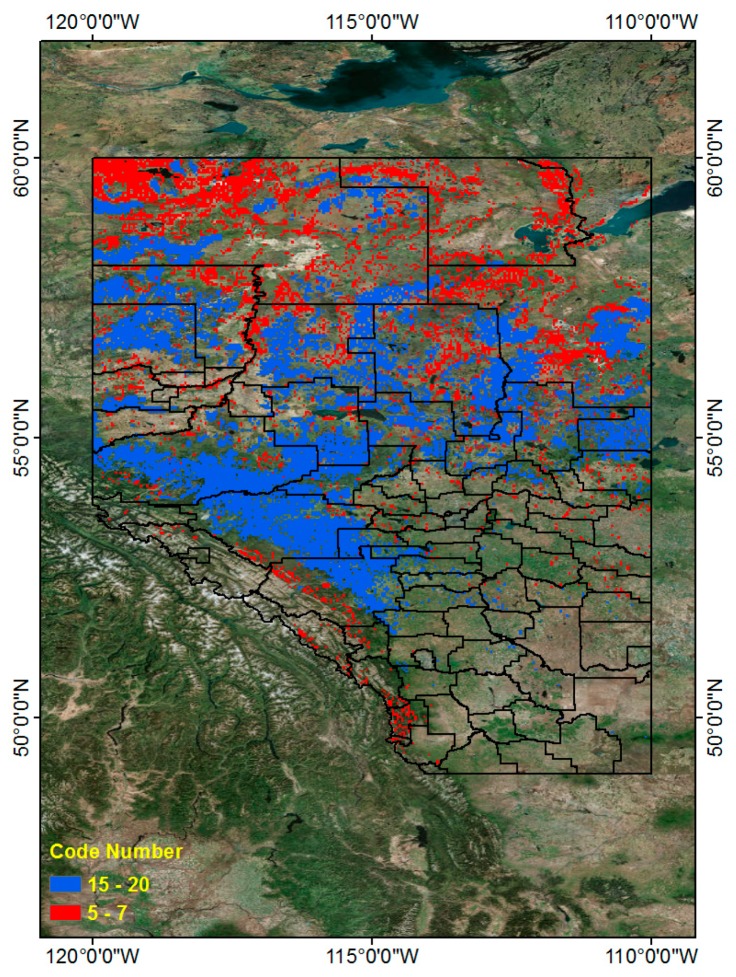
Combined wildfire location (Code Number 5–7 and Code Number 15–20).

Using Table 12, we can find PI higher than 1 in 9 code numbers (5, 6, 7, 15, 16, 17, 18, 19 and 20) out of 20 classes. As mentioned above, we can classify these into two groups. The first includes those regions with a relatively low frequency of CG lightning, yet a relatively high frequency of wildfires, and the second includes those regions with a relatively high incidence of both lightning strikes and wildfires caused by lightning. We determined that code numbers 5, 6 and 7 define the first group and that code numbers 15–20 define the second. We present these two groups (Red and Blue) in Figure 17 to illustrate two relatively higher risk wildfire regions in the PI graph. The combined Red and Blue regions are the final wildfire hazardous regions (Figure 18).

Using association rule mining techniques, we analyzed CG lightning frequent patterns and their related land properties and generated a hazard map based on the patterns. We tried to find a relationship between CG lightning frequent patterns and wildfires caused by CG lightning. We found that some regions have a high incidence of both CG lightning and wildfires (Blue color regions), but others combine a lower incidence of CG lightning with a relatively high incidence of wildfires (Red color regions). These results can be utilized to locate regions of high wildfire risk and to identify their related land properties, using association rule mining results to be managed and prepared for the wildfire hazard in Alberta.

## 8. Discussion and Conclusions 

We investigated the characteristics of CG lightning over Alberta, Canada, by lightning date between 2010 and 2016. We implemented a hotspot analysis to find the regions with high frequency CG lightning strikes clustered together. Generally, hotspot regions are located in central, central east and south central regions of the study area. A total of 93.8% of annual lightning occurs in warm months (June to August) and the daily lightning frequency is influenced by the diurnal heating cycle. We used the association rule mining technique (CFP-Growth++ algorithm) to investigate frequent CG lightning patterns. The frequent CG lightning patterns were verified by a similarity measurement to check the patterns’ consistency. We verified the CG lightning hazard map for 2010–2014 by comparing it to unprocessed independent raw CG lightning data from 2015 to 2016. The resulting similarity coefficient values showed a high correlation throughout the study period. The actual CG lightning generally flashed more in higher risk regions in the lightning hazard map. Most wildfires in Alberta (approximately 93%) occur in Forests, Wetland Forests, and Wetland Shrub areas. We found two distinct areas of interest: frequent wildfire regions with a high frequency of lightning, and frequent wildfire regions with a low frequency of lightning. Further, the preference index (PI) revealed locations where wildfires occurred more frequently than in other class regions. One potential application of this research is to estimate wildfire hazard areas against CG lightning hazard maps and frequency data for specific land use types. There are limitations in this study. First, we analyzed only seven years of CG lightning data. Analyzing additional years of CG lightning data would provide more accurate results. This study considered a limited number of land properties (i.e., Elevation, Slope, Land Uses, and Soil Types). Including more land properties in the study would enhance the accuracy of our results. In addition, there may be other factors that we did not consider, such as Convective Available Potential Energy (CAPE), moisture content, surface temperature, etc. Accuracy and reliability of the results could be improved by adapting other data mining techniques as well.

## Figures and Tables

**Table 1:** Percentage of average lightning counts by month between 2010 and 2014.

Month	%	Total
December	0.0004	
January	0.0011	0.0019%
February	0.0004	
March	0.0188	
April	0.2673	3.3875%
May	3.1014	
June	18.8760	
July	45.6683	93.8473%
August	29.3030	
September	2.5636	
October	0.1893	2.7633%
November	0.0105	

**Table 2:** CG lightning frequent patterns on four land properties (2010–2014).

**2010 High Risk**				
**Item 1 (Elevation)**	**Item 2 (Slope)**	**Item 3 (Land Uses)**	**Item 4 (Soil Types)**	**Support Count**
5	12	25	39	6210
5	11	29	44	6117
3	13	25	39	5638
2	11	26	38	4059
**2010 Risk**				
6	12	25	39	3432
6	13	25	39	3230
4	11	29	43	3042
3	11	26	39	2904
4	11	30	43	2888
4	11	30	44	2474
3	11	29	43	2313
4	12	30	44	2283
4	12	30	43	2223
1	11	33	38	2132
6	14	25	39	2049
1	11	25	37	2039
6	11	25	39	1947
3	11	24	39	1875
5	14	25	39	1854
4	12	29	43	1840
3	14	25	39	1743
7	14	25	39	1696
2	11	25	37	1673
7	13	25	39	1662
**2011 High Risk**				
**Item 1 (Elevation)**	**Item 2 (Slope)**	**Item 3 (Land Uses)**	**Item 4 (Soil Types)**	**Support Count**
5	12	25	39	7450
5	11	29	44	6269
3	13	25	39	6126
5	13	25	39	5502
2	11	26	38	4738
6	12	25	39	4437
6	13	25	39	4109
**2011 Risk**				
3	11	26	39	3328
6	11	25	39	2628
6	14	25	39	2566
5	14	25	39	2413
4	11	30	44	2410
4	11	30	43	2304
4	12	30	44	2250
3	11	29	43	2205
4	11	29	43	1993
**2012 High Risk**				
**Item 1 (Elevation)**	**Item 2 (Slope)**	**Item 3 (Land Uses)**	**Item 4 (Soil Types)**	**Support Count**
5	12	25	39	10,321
5	11	29	44	8530
5	13	25	39	7769
3	13	25	39	7153
6	12	25	39	6702
6	13	25	39	6268
**2012 Risk**				
2	11	26	38	5101
3	11	26	39	4556
6	11	25	39	3929
6	14	25	39	3887
5	14	25	39	3628
**2013 High Risk**				
**Item 1 (Elevation)**	**Item 2 (Slope)**	**Item 3 (Land Uses)**	**Item 4 (Soil Types)**	**Support Count**
5	11	29	44	11,829
5	12	25	39	7880
**2013 Risk**				
4	11	30	44	4783
6	12	25	39	4687
2	11	26	38	4592
6	13	25	39	4552
4	12	30	44	4329
3	11	26	39	3371
4	11	30	43	3351
**2014 High Risk**				
**Item 1 (Elevation)**	**Item 2 (Slope)**	**Item 3 (Land Uses)**	**Item 4 (Soil Types)**	**Support Count**
5	11	29	44	10,570
5	12	25	39	8155
**2014 Risk**				
2	11	26	38	5675
4	11	30	43	5402
4	11	30	44	5224
4	11	29	43	4792
4	12	30	44	4712
6	12	25	39	4255
3	11	26	39	4107
6	13	25	39	4064
1	11	33	38	3886
4	12	30	43	3786
1	11	25	37	3646

**Table 3:** Sub-set frequent pattern result 2010 (left) and 2011 (right).

2010					2011				
Var1 (Elevation)	Var2 (Slope)	Var3 (Land Uses)	Var4 (Soil Types)	Support Counts	Var1 (Elevation)	Var2 (Slope)	Var3 (Land Uses)	Var4 (Soil Types)	Support Counts
5	12	25	39	6210	5	12	25	39	7450
5	11	29	44	6117	5	11	29	44	6269
3	13	25	39	5638	3	13	25	39	6126
2	11	26	38	4059	5	13	25	39	5502
6	12	25	39	3432	2	11	26	38	4738
6	13	25	39	3230	6	12	25	39	4437

**Table 4:** Ranked frequent pattern result of 2010 (left) and 2011 (right).

2010					2011				
Var1 (Elevation)	Var2 (Slope)	Var3 (Land Uses)	Var4 (Soil Types)	Support Counts	Var1 (Elevation)	Var2 (Slope)	Var3 (Land Uses)	Var4 (Soil Types)	Support Counts
5	12	25	39	Rank 1	5	12	25	39	Rank 1
5	11	29	44	Rank 2	5	11	29	44	Rank 2
3	13	25	39	Rank 3	3	13	25	39	Rank 3
2	11	26	38	Rank 4	5	13	25	39	Rank 4
6	12	25	39	Rank 5	2	11	26	38	Rank 5
6	13	25	39	Rank 6	6	12	25	39	Rank 6

**Table 5:** Similarity coefficient by modified Best Match algorithm with differrent *p*-values.

*p* = 1.0		2010	2011	2012	2013	2014
	2010	1	0.877	0.869	0.867	0.871
	2011		1	0.878	0.854	0.862
	2012			1	0.877	0.863
	2013				1	0.857
	2014					1
*p* = 0.8		2010	2011	2012	2013	2014
	2010	1	0.887	0.88	0.878	0.881
	2011		1	0.889	0.864	0.872
	2012			1	0.886	0.875
	2013				1	0.867
	2014					1
*p* = 0.6		2010	2011	2012	2013	2014
	2010	1	0.897	0.891	0.889	0.891
	2011		1	0.90	0.875	0.882
	2012			1	0.895	0.886
	2013				1	0.877
	2014					1
*p* = 0.4		2010	2011	2012	2013	2014
	2010	1	0.908	0.903	0.901	0.902
	2011		1	0.912	0.887	0.893
	2012			1	0.904	0.898
	2013				1	0.888
	2014					1
*p* = 0.2		2010	2011	2012	2013	2014
	2010	1	0.919	0.915	0.912	0.913
	2011		1	0.924	0.899	0.904
	2012			1	0.913	0.911
	2013				1	0.898
	2014					1

**Table 6:** CG-lightning frequent patterns on four land properties from 2010 to 2014 and from 2015 to 2016.

**2010–2014 High Risk**				
**Item 1 (Elevation)**	**Item 2 (Slope)**	**Item 3 (Land Uses)**	**Item 4 (Soil Types)**	**Support Count**
5	11	29	44	43,315
5	12	25	39	40,016
**2010–2014 Risk**				
2	11	26	38	24,165
6	12	25	39	23,513
6	13	25	39	22,223
3	11	26	39	18,266
4	11	30	44	17,730
4	11	30	43	16,503
4	12	30	44	16,196
4	11	29	43	15,379
**2015–2016 High Risk**				
**Item 1 (Elevation)**	**Item 2 (Slope)**	**Item 3 (Land Uses)**	**Item 4 (Soil Types)**	**Support Count**
5	11	29	44	26,055
5	12	25	39	19,813
**2015–2016 Risk**				
6	12	25	39	11,956
6	13	25	39	10,779
4	11	30	44	9754
2	11	26	38	9443
4	12	30	44	9072
3	11	26	39	9020
4	11	29	43	8279
4	11	30	43	8165
3	11	29	43	7033
6	11	25	39	7000
6	14	25	39	6909
5	14	25	39	6672

**Table 7:** Similarity coefficient results using the Modified Best Match algorithm with different *p*-values (*p* = 1, 0.8, 0.6, 0.4 and 0.2) between 2010–2014 and 2015–2016.

	*p* = 1	*p* = 0.8	*p* = 0.6	*p* = 0.4	*p* = 0.2
Similarity Coefficient Value (2010–2014 vs. 2015–2016)	0.920	0.927	0.933	0.939	0.946

**Table 8:** The divided classes’ value ranges of the hazard map (2010–2014) and the area size percentage of total frequency of CG lightning (2015–2016).

Class Code Number	Hazard Map Value Ranges and Percentage of Risky Levels	Area Size Percentage over Total
1	15–769.41 (0–1.77%)	5%
2	769.41–1229.74 (1.77–2.83%)	5%
3	1229.74–1623.63 (2.83–3.74%)	5%
4	1623.63–2046.80 (3.74–4.72%)	5%
5	2046.80–2650.59 (4.72–6.11%)	5%
6	2650.59–3255.78 (6.11–7.51%)	5%
7	3255.78–3961.87 (7.51–9.14%)	5%
8	3961.87–4794.87 (9.14–11.06%)	5%
9	4794.87–5898.45 (11.06–13.61%)	5%
10	5898.45–7202.03 (13.61–16.62%)	5%
11	7202.03–8243.86 (16.62–19.03%)	5%
12	8243.86–9339.75 (19.03–21.56%)	5%
13	9339.75–10,097.06 (21.56–23.31%)	5%
14	10,097.06–11,423.83 (23.31–26.37%)	5%
15	11,423.83–13,946.35 (26.37–32.19%)	5%
16	13,946.35–16,222.81 (32.19–37.45%)	5%
17	16,222.81–18,223.84 (37.45–42.07%)	5%
18	18,223.84–23,756.30 (42.07–54.84%)	5%
19	23,756.30–36,759.70 (54.84–84.86%)	5%
20	36,759.70–43,315 (84.86–100%)	5%

**Table 9:** The actual CG lightning (2015–2016) for each class of CG lightning hazard map (2010–2014).

Class Code Number	Hazard Map Value Ranges and Percentage of Risky Levels	Raw CG Lightning Counts (2015–2016)	Percentage of Raw CG Lightning Count of Total (2015–2016)
1	15–769.41 (0–1.77%)	39,461	4%
2	769.41–1229.74 (1.77–2.83%)	36,632	4%
3	1229.74–1623.63 (2.83–3.74%)	40,946	4%
4	1623.63–2046.80 (3.74–4.72%)	36,169	4%
5	2046.80–2650.59 (4.72–6.11%)	43,331	5%
6	2650.59–3255.78 (6.11–7.51%)	38,394	4%
7	3255.78–3961.87 (7.51–9.14%)	37,341	4%
8	3961.87–4794.87 (9.14–11.06%)	38,791	4%
9	4794.87–5898.45 (11.06–13.61%)	40,591	4%
10	5898.45–7202.03 (13.61–16.62%)	45,861	5%
11	7202.03–8243.86 (16.62–19.03%)	49,284	5%
12	8243.86–9339.75 (19.03–21.56%)	48,505	5%
13	9339.75–10,097.06 (21.56–23.31%)	41,939	4%
14	10,097.06–11,423.83 (23.31–26.37%)	44,127	5%
15	11,423.83–13,946.35 (26.37–32.19%)	57,014	6%
16	13,946.35–16,222.81 (32.19–37.45%)	57,621	6%
17	16,222.81–18,223.84 (37.45–42.07%)	58,592	6%
18	18,223.84–23,756.30 (42.07–54.84%)	61,979	6%
19	23,756.30–36,759.70 (54.84–84.86%)	66,609	7%
20	36,759.70–43,315 (84.86–100%)	70,793	7%
**Sum**		**953,980**	**100%**

**Table 10:** Wildfires caused by CG lightning on land use types in Alberta (2010–2014).

Land Uses	Count of Wildfire Caused by Lightning	Percentage of the Wildfire Count of Total
Unclassified	0	0%
Settlement	7	0.002%
Roads	10	0.41%
Water	32	1.33%
Forest	1383	57.86%
Forest Wetland	566	23.68%
Trees	6	0.25%
Treed Wetland	28	1.17%
Cropland	14	0.58%
Grassland Managed	2	0.08%
Grassland Unmanaged	2	0.08%
Wetland	6	0.25%
Wetland Shrub	283	11.84%
Wetland Herb	46	1.92%
Other Land	5	0.2%
**Sum**	2390	100%

**Table 11:** CG lightning hazard map classes and wildfire frequency within a class.

Ranges	Code Number	Code Size	Percentage of Total for Each Code	Wildfire Counts (2010–2014)	Normalized Wildfire Counts (2010–2014)
15–769.41 (1.77%)	1	1808	5%	92	0.05
769.41–1229.74 (2.83%)	2	1643	4%	52	0.031
1229.74–1623.63 (3.74%)	3	1544	4%	58	0.037
1623.63–2046.80 (4.72%)	4	1794	5%	87	0.048
2046.80–2650.59 (6.11%)	5	1745	5%	132	0.075
2650.59–3255.78 (7.51%)	6	1990	5%	155	0.077
3255.78–3961.87 (9.14%)	7	2048	5%	128	0.062
3961.87–4794.87 (11.06%)	8	1835	5%	91	0.049
4794.87–5898.45 (13.61%)	9	2195	6%	121	0.055
5898.45–7202.03 (16.62%)	10	1978	5%	106	0.053
7202.03–8243.86 (19.03%)	11	1668	4%	84	0.050
8243.86–9339.75 (21.56%)	12	2327	6%	114	0.048
9339.75–10,097.06 (23.31%)	13	2335	6%	101	0.043
10,097.06–11,423.83 (26.37%)	14	2233	6%	123	0.055
11,423.83–13,946.35 (32.19%)	15	1758	5%	122	0.069
13,946.35–16,222.81 (37.45%)	16	1645	4%	122	0.074
16,222.81–18,223.84 (42.07%)	17	1535	4%	134	0.087
18,223.84–23,756.30 (54.84%)	18	2482	7%	205	0.082
23,756.30–36,759.70 (84.86%)	19	2092	6%	155	0.074
36,759.70–43,315 (100%)	20	711	2%	49	0.068

**Table 12:** Preference Index (PI) for each code.

Code Number	PI	Code Number	PI
1	0.852	11	0.843
2	0.53	12	0.82
3	0.629	13	0.724
4	0.812	14	0.922
5	1.266	15	1.162
6	1.304	16	1.242
7	1.046	17	1.462
8	0.83	18	1.383
9	0.923	19	1.24
10	0.897	20	1.154

## Appendix A

The information of code label number for the classified land properties.

**Table A1:** Information of code label number of elevation.

Item 1 (Global Digital Elevation Model)	
Code	Label (Meter)
1	168–408
2	408–588
3	588–722
4	722–867
5	867–1054
6	1054–1289
7	1289–1587
8	1587–1943
9	1943–2344
10	2344–3709

**Table A2:** Information of code label number of slope.

Item 2 (Slope)	
Code	Label (Degree)
11	0–1.704632
12	1.704632–3.634515
13	3.634515–6.265699
14	6.265699–10.070405
15	10.070405–15.106709
16	15.106709–21.327051
17	21.327051–28.527229
18	28.527229–36.822903
19	36.822903–49.206623
20	49.206623–85.246559

**Table A3:** Information of code label number of land uses.

Item 3 (Land Uses)		
Code	Label	Definition
21	Unclassified	Areas not classified due to clouds
22	Settlement	Built-up and urban
23	Roads	Primary, secondary and tertiary
24	Water	Natural and human-made
25	Forest	Treed areas >1 ha in size
26	Forest Wetland	Wetland with forest cover
27	Trees	Treed areas <1 ha in size
28	Treed Wetland	Wetland with tree cover
29	Cropland	Annual and perennial
30	Grassland Managed	Natural grass and shrubs used for cattle grazing
31	Grassland Unmanaged	Natural grass and shrubs with no apparent use (forest openings, alpine meadows, tundra, etc.)
32	Wetland	Undifferentiated wetland
33	Wetland Shrub	Wetland with shrub cover
34	Wetland Herb	Wetland with grass cover
35	Other Land	Rock, beaches, ice, barren land

**Table A4:** Information of code label number of soil types.

Item 4 (Soil Types)	
Code	Label
36	Cryosolic
37	Brunisolic
38	Organic
39	Luvisolic
40	Gleysolic
41	Misc. Bedrocks or Water
42	Regosolic
43	Solonetzic
44	Chernozemic
45	Vertisolic
Reference: http://sis.agr.gc.ca/cansis/soils/provinces.html **Soil Subgroup**: Identifies the soil subgroup according to the Canadian System of Soil Classification (http://www.soilsofcanada.ca/orders/index.php) [38].	
